# Tongue necrosis in a patient with cranial arteritis

**DOI:** 10.1016/S1808-8694(15)30135-X

**Published:** 2015-10-19

**Authors:** Gerson Schulz Maahs, Daniela Dias Fabricio

**Affiliations:** a PhD in Surgery, M.S. in Medicine. Otorhinolaryngology and Head and Neck Surgery Consultant. Professor at the Medical Residency; b Otorhinolaryngologist, colaborator at the Otorhinolaryngology - Head and Neck Surgery Department of the São Lucas Hospital - PUCRS. Serviço de Otorrinolaringologia e Cirurgia de Cabeça e Pescoço do Hospital São Lucas da PUCRS, Porto Alegre RS

**Keywords:** cranial arteritis, horton’s disease, temporal arteritis, tongue infarction, tongue necrosis

## INTRODUCTION

Cranial Arteritis (CA) is a giant cells vasculitis of unknown cause, which preferentially involves the extracranial branches of the carotid artery. It is the most common systemic vasculitis in adults ^1^.

Age is its major risk factor, and it affects patients above 50 years of age.

CA’s classic manifestations are headache, mandibular claudication, rheumatic polymyalgia and visual symptoms^1^, ^2^, ^3^. Headache is the most common finding in about 70% of the patients affected. Rheumatic polymyalgia is usually seen as the systemic stage in CA, characterized by pain in the shoulders, neck and hips.

Visual symptoms such as vision loss and diplopia happen to one third of the patients. Blindness is the most feared complication. Systemic symptoms such as weight loss, fever and arthralgia are common.

In 40% of the patients there are atypical otorhinolaryngological symptoms such as dry cough, sore throat, dysphonia, neck pain and tongue involvement^1^, ^4^. Tongue involvement is not uncommon, the main complaints are paresthesias, pain, glossitis and tongue claudication. Despite all of these, tongue necrosis and ischemia are rare events^5^.

Diagnosis is based on clinical history, physical exam and an increase in the erythrocyte sedimentation rate (ESR), which is usually higher than 100 mm/h^1^, ^2^, ^3^, ^4^, ^5^. In the patient’s exam we can find anomalies in the temporal artery such as pulsation increase or interruption. Diagnosis is confirmed by temporal bone biopsy, which shows mononuclear infiltration or granulomatous inflammation.

## CLINICAL CASE

**Figure 1:**
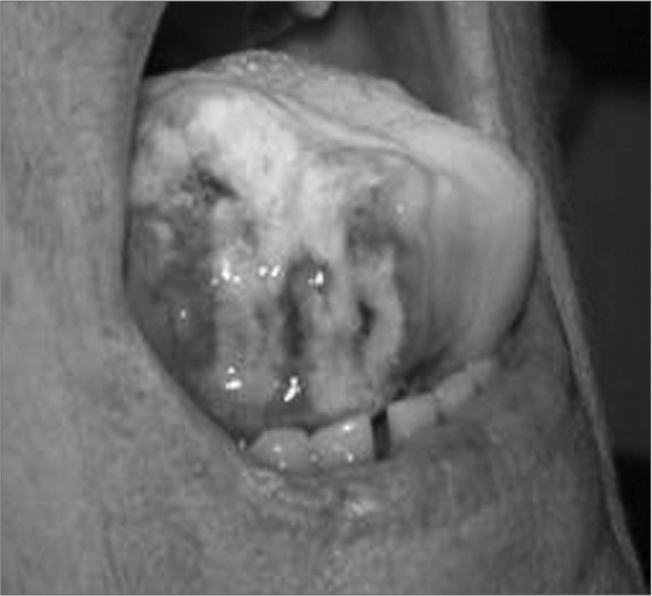
Tongue necrosis.

A 68 year old Caucasian female, started having pain in her right temporal region and mandible. Later on, she presented with intense tongue pain, which in 30 hours evolved to tongue necrosis and ulceration in the right side latero-anterior region, with considerable loss of tongue tissue (Figure 1).

The right temporal artery was thickened. The ESR was of 113 mm/h. We started her on 60mg of prednisone and 100mg of azathioprine. Clinical response came after three months, and was very satisfactory, with regeneration of the tongue tissue.

## DISCUSSION

Tongue necrosis is a rare occurrence in cranial arteritis. It happens through the stenosis and occlusion of the lingual and contralateral arteries. Tongue claudication and pain during swallowing are important warning signs in order to avoid its evolution towards tongue ischemia and necrosis. Once the ischemic process starts, it seems to progressively evolve towards tongue necrosis. In half of the patients, the necrosis is symmetrical in both sides of the tongue, in 20% the tongue dorsum is involved; and in 25% only the tongue tip is involved. In our case, the necrosis site was very uncommon, being assymetrical and involving the right side latero-anterior portion of the tongue.

Some authors have described the onset of ischemia related to the use of ergotamine^5^, ^6^, and self medication with this drug is usual in order to treat headache - a common symptom. This would happen because of a vasospasm caused by the drug in question, contributing to the arterial occlusion.

Tongue biopsy is usually non-specific and is not indicated.

The treatment of choice for CA is high doses of steroids, which may be associated to other immunosuppressants such as azathyoprine and methotrexate.

## FINAL REMARKS

Although reported in the literature, tongue necrosis is not common. The otorhinolaryngologist must be familiarized with this pathology and its atypical manifestations in order to properly prevent its occurrence and also be able to treat its complications.
